# Multi-Drug Resistant Bacteria as Aetiological Factors of Infections in a Tertiary Multidisciplinary Hospital in Poland

**DOI:** 10.3390/antibiotics10101232

**Published:** 2021-10-10

**Authors:** Sławomir Poletajew, Katarzyna Pawlik, Anna Bonder-Nowicka, Artur Pakuszewski, Łukasz Nyk, Piotr Kryst

**Affiliations:** 1 Second Department of Urology, Centre of Postgraduate Medical Education, 80 Cegłowska St., 00809 Warsaw, Poland; anna.bonder-nowicka@cmkp.edu.pl (A.B.-N.); apa13@wp.pl (A.P.); ukinyk@poczta.fm (Ł.N.); piotr.kryst@cmkp.edu.pl (P.K.); 2 Infection Control Team, Bielanski Hospital, 80 Cegłowska St., 00809 Warsaw, Poland; katarzyna.pawlik@bielanski.med.pl

**Keywords:** antibiotics, antibiotic resistance, antimicrobial drug resistance, beta-lactamase resistance, hospital infections, multidrug resistance

## Abstract

Global and local initiatives were recently undertaken to reduce the burden of antibiotic resistance. The aim of the study was to describe the incidence and the aetiology of bacterial infections among hospitalized patients with special attention paid to the multidrug resistant (MDR) bacteria. This retrospective study was based on prospectively collected data from 150,529 consecutive patients hospitalized in a tertiary multidisciplinary hospital in the years 2017–2019. All consecutive microbiological tests from any biological material performed in the analyzed period were included. Microbiological screening tests (*n* = 10,677) were excluded. The analysis was focused on aetiological factors of bacterial infections, especially the incidence of MDR bacteria and mechanisms of antibiotic resistance. There were 58,789 microbiological tests performed in the analyzed period. The highest testing rate was noticed for intensive care unit (mean of 3.1 tests per one patient), followed by neonatal intensive care unit (2.7), internal medicine (1.9), pediatrics (1.8), and urology (1.2). Among 58,789 tests, 7690 (13.1%) were positive. MDR bacteria were responsible for 1783 infections (23.2%). The most common antibiotic resistance mechanism reported was ESBL production by *Klebsiella* spp. or *Escherichia coli* or *Enterobacter* spp. isolates (47.3% of all MDR cases). ESBL cases were followed by MRSA (14.7%), VRE (14.2%) and MBL producing *Klebsiella* spp. (5.6%). Among all infections caused by MDR bacteria, 1175 (65.9%) were diagnosed after 72 h of hospitalization (hospital-acquired infections). Apart from AmpC and ESBL producing *Escherichia coli*, all MDR bacteria were significantly more common in hospital-acquired infection. MDR bacteria are aetiological factors of a significant portion of infections in hospitalized patients with no remarkable change in the incidence in recent years. Production of ESBL is the most common mechanism of antibiotic resistance and should be regarded as one of the most urgent problems in clinical microbiology.

## 1. Introduction

Bacterial infections belong the most common and disabling diseases [1]. Moreover, today they are closely related to the phenomenon of drug resistance, namely antibiotic resistance. According to data from EARS-Net, resistance to at least one antimicrobial therapeutic group in Europe exceeded 57% for *Escherichia coli* (*E. coli*) isolates (aminopenicillins 57.1%, fluoroquinolones 23.8%, third-generation cephalosporins 15.1%, and aminoglycosides 10.8%) and 36% for *Klebsiella pneumoniae* (*K. pneumoniae*) isolates (third-generation cephalosporins 31.3%, fluoroquinolones 31.2%, aminoglycosides 22.3%, carbapenems 7.9%; intrinsically resistant to aminopenicillins). In 2019, the percentage of combined resistance, measured as resistance to fluoroquinolones, third-generation cephalosporins, and aminoglycosides (multidrug resistant strains) was reported to be 5.9% for *E. coli*, 19.3% for *K. pneumoniae*, 29.7% for *Acinetobacter* spp., and 12.1% for *Pseudomonas aeruginosa* (*P. aeruginosa*). Simultaneously, the incidence of meticillin-resistant *Staphylococcus aureus* (MRSA) was 15.5%, and the incidence of vancomycin-resistant *Enterococcus faecium* (VRE) was 18.3%. Resistance to vancomycin among *Enterococcus faecalis* was low [2]. As reported by the World Health Organization, antibiotic resistance is associated with severe negative clinical and economic consequences [3]. At the same time, the incidence of bacteria resistant to antibiotics increases [4,5,6]. Today, there are regions in the world where multidrug resistant (MDR) bacteria are responsible for of >75% of infections in hospitalized patients [7,8,9].

Local and global initiatives have been initiated to reduce the burden of antibiotic resistance in last years. According to recent data from EARS-Net and ESAC-Net study groups, these actions resulted in reduction of antibiotic use and stabilization of the antibiotic resistance incidence [10]. However, such outcomes are not universal. In Poland, the rate of MDR bacteria is increasing along with the use of carbapenems [11]. Next to continuation of the efforts to save antibiotics, detailed and updated data on the incidence and resistance patterns in bacterial infections are needed.

The aim of the study was to describe the incidence and the aetiology of bacterial infections among hospitalized patients with special attention paid to the MDR bacteria.

## 2. Results

There were 58,789 microbiological tests performed in the analyzed period. The number of tests did not differ significantly between years 2017, 2018, and 2019 (19,838 vs. 18,123 vs. 20,828). Table 1 presents number of tests performed in different hospital departments. The highest testing rate was noticed for intensive care unit (mean of 3.09 tests per one patient), followed by neonatal intensive care unit (2.72), internal medicine (1.87), pediatrics (1.77), and urology (1.2). Blood cultures and urine cultures accounted for 50.1% of performed microbiological tests (25.7% and 24.4%, respectively).

Among 58,789 tests, 7690 (13.1%) were positive. Table 2 presents aetiological factors of reported infections depending on the medical specialty of the hospital department. MDR bacteria were responsible for 1783 infections (23.2%). The most common antibiotic resistance mechanism reported was the production of extended-spectrum beta-lactamases (ESBL) by *Klebsiella* spp. or *E. coli* or *Enterobacter* spp. isolates (47.3% of all MDR cases). ESBL cases were followed by MRSA (14.7%), vancomycin-resistant *Enterococcus* (VRE) (14.2%), and *Klebsiella* spp. producing metallo-beta-lactamase (MBL) (5.6%).

Among all infections caused by MDR bacteria, 1175 (65.9%) were diagnosed after 72 h of hospitalization (hospital-acquired infections). Table 3 presents a list of MDR bacteria as aetiological factors of infections together with data on the rate of hospital-acquired infections. Apart from AmpC and ESBL producing *E. coli*, all MDR bacteria were significantly more common in hospital-acquired infection. Table 4 presents a subanalysis of data for different hospital departments. One can see a trend for higher percentage of MDR strains in intensive care unit. Finally, we observed the increase in MRSA incidence and decrease in VRE incidence over time in the analyzed period, with no clinically significant difference in the rate of infections caused by other MDR bacteria (Table 5).

## 3. Discussion

We conducted an analysis of the incidence and the aetiology of bacterial infections among representative sample of consecutive hospitalized patients. There are several messages coming from this study. 

First, we found that Gram negative bacteria remain the main aetiology of infections, with *E. coli* and *Klebsiella* spp. being by far the most commonly isolated bacteria (48% of all infections). Apart from intensive care units, such aetiology is stably observed for a long time [12,13,14].

Second, regarding MDR bacteria, the most common mechanism noticed in this study was the production of ESBL by *Klebsiella* spp., *E. coli* or *Enterobacter* (47% of all MDR cases). This observation became standard and was also reported by Mataj et al. and Wang et al. in Asia [15,16], Acma et al. in America [17], and Arana et al. in Europe [18].

Third, among patients with hospital-acquired MDR infections, the absolute number of cases was still the highest for ESBL producing *Klebsiella* spp. and *Enterobacter* spp.; however, a high rate of VRE and MBL producing *Klebsiella* was also reported (79% of these infections were hospital-acquired). A high rate of ESBL bacterial strains results in increasing clinical pressure to use carbapenems, as well as increasing risk of persistence of carbapenemase producing *Enterobacteriaceae* (CPE) and transmission. AmpC beta-lactamases and ESBL producing *E. coli* were the only MDR bacteria that were more likely to be acquired before admission to hospital (71.4% and 59.6%, respectively). Unfortunately, other drug-resistant microorganisms were also found on admission to the hospital, especially ESBL producing *Klebsiella* spp. and MRSA, which were community-based in 39% and 27% of infections, respectively. This situation requires deep analysis of the risk factors for the multi-drug-resistant infection, including a history of a hospitalization, previous antibiotic therapy, and others. Based on this analysis, the extension of screening tests for the early identification of patients infected/colonized with MDR bacteria would be of utmost importance to limit the spread of MDR bacteria in the hospital and to implement effective upfront antibiotic therapy. In face of the high incidence of MDR bacteria among patients with community-based infections, there is a need to implement rapid diagnostic tests to early diagnose drug resistance. This should be considered in all patients, critically-ill patients, or at least in cases of positive blood cultures and cultured bacterial colonies. Examples of such tests are the carba NP test, identification of resistance mechanisms by mass spectrometry in positive blood samples or the most developing molecular methods such as real-time PCR (diagnosis of sepsis, respiratory tract infections), DNA microarrays, or a diagnostic method utilizing miniaturized magnetic resonance technology [19].

Finally, when comparing three consecutive years included into this analysis, we did notice clinically significant change in the rate of infections caused by MDR bacteria regarding MRSA (increase from 25% to 35%), *E. faecium* (decrease from 65% to 38%) and *E. faecalis* (decrease from 11% to 6%) strains. The decrease in rate of MDR-related infections was probably a result of the implementation of supervision over the hospital environment and the implemented strategy to combat *Clostridioides difficile* in the hospital. Interestingly, a peak of incidence of infections caused by New Delhi type carbapenemase (MBL) producing *K. pneumoniae* was noticed in the analysed period. However, after they accounted for 7.9% of infections in 2017, these percentage increased up to 14.8% in 2018 to finally decrease to 7.8% in 2019 (Table 5). This decrease is related to the implementation of a multi-module strategy to prevent the spread of CPE implemented by all municipal hospitals in Warsaw (described on the website of the National Antibiotic Protection Program) [20]. This multi-module strategy was implemented in ten municipal hospitals and the long-term care facilities in Warsaw. Moreover, basic rules to prevent and control CPE infections were adopted in all healthcare centers of the city, regardless of the presence of CPE. In brief, all centers were instructed about the indications for screening tests, methods of CPE infection prophylaxis, and management in case of CPE infection or colonization. Procedures were regularly controlled by internal and external audits.

However, it should be noted that the emergence of carbapenem-resistant strains in our hospital resulted in the increased colistin consumption. Since 2018, the occurrence of acquired resistance to colistin has been observed among Enterobacterales, in particular *K. pneumoniae* and *E. coli*. Resistance was detected by phenotypic methods, and each resistant strain was sent to a reference center (KORLD) for confirmation. Unfortunately, data on the molecular resistance mechanism is not available in this analysis and it remains to be determined whether it was a *mcr* gene associated resistance or chromosomal resistance. Due to the coexistence of colistin resistance with the production of ESBLs and/or carbapenemases, we were not able to distinguish a separate group of colistin-resistant strains as a part of microbiological monitoring. However, this phenomenon requires close monitoring as a part of the analysis of cumulative antibiograms, including the use of polymyxins in the hospital. Resistance to colistin in Enterobacterales has been widely reported worldwide, including Europe. The emerging outbreaks of resistant strains are the reason for the limited possibilities of empirical therapy in severe infections in hospital wards with CPE [21].

The significance of MDR bacterial infections is well known and can be considered at least in two aspects, namely clinical and economical. The MDR aetiology of infection, especially ESBL Enterobacterales, was shown to be associated with the prolonged hospitalization time and increased risk of death [22,23,24,25]. In the recent meta-analysis, the presence of cephalosporin-resistant *E. coli* was associated with over 2-fold higher 30-day mortality [26]. The inverse relation of the spectrum of resistance to antimicrobials and the survival was recently demonstrated by Santoro et al. [27].

From an economical point of view, treatment of MDR bacterial infections is 1.6-fold more expensive [28]. Moreover, it is associated with patients’ temporal disability. Cephalosporin-resistant *E. coli* and *K. pneumoniae*, mainly ESBL producing isolates, are associated with a significant number of disability-adjusted life-years (DALYs). Based on a population-level modeling analysis from Cassini et al., median number of DALYs per 10,000 population is estimated for 37 and 23 for cephalosporin-resistant *E. coli* and *K. pneumoniae*, respectively [29].

While in general the data presented in this study is in line with available European data, interesting is the local increase in the rate of MRSA isolation, which is contrary to general European observations reported by European Centre for Disease Prevention and Control [2]. Similar increasing trend was recently described by another Polish research team. Moreover, Kot et al. that the ratio of MDR MRSA isolates is increasing and exceed 92%. Authors concluded about rapid acquisition of new antimicrobial resistance determinants by MRSA isolates in the hospital environment [30]. This needs further concern among clinicians and appropriate action to stop the trend.

Every fifth bacterial infection diagnosed in the analyzed period was of MDR aetiology. There is a general increasing trend of MDR bacterial infections [4,5,6], while the rate of MDR infections can be as high as 90% in Asia or Africa [7,8]. Differences in the rate of MDR infections between countries and regions are well known. They were described by Dornbusch et al. for European regions in 1998 [31]. In Europe, lower rate of MDR bacteria in Nordic hospitals is still observed and was recently confirmed by Moller et al. [32]. Kock et al. indicated that these differences can result from differences in the density of in-patient care, density of hospital personnel, rate of ambulatory surgery, or average length of hospitalization. All these parameters can vary significantly even between neighboring countries [33]. However, significant differences between hospitals are described also within one country, as Kreidl et al. described for two hospitals in Austria [34]. These phenomena can be both a reason and a consequence of different antimicrobial consumption observed in different world regions [35].

Strengths of or study are the inclusion of high number of consecutive patients, prospective collection of data and separate analysis for different medical specialities and years. However, our study is not free from limitations. First, we did not analyze separately different clinical manifestations of infections. Second, data on the impact of MDR bacteria on clinical course was not reported. Finally, data on colistin resistance cannot be presented as, according to local policy, susceptibility to colistin is tested only in carbapenem-resistant strains.

## 4. Material and Methods

In the years 2017–2019, 150,529 patients were hospitalized in a tertiary multidisciplinary hospital. This retrospective study is based on prospectively collected data from central microbiological laboratory. All consecutive microbiological tests from any biological material performed in the analyzed period were included. Duplicate isolates, namely further isolates of the same pathogen with indistinguishable antibiograms obtained from the same patient, were excluded. Also, microbiological screening tests (n = 10,677) were excluded. All strains were identified by mass spectrometry (MALDI-TOF Microflex LT, Bruker; Billerica, Massachusetts, USA). Drug susceptibility tests were carried out using the disc diffusion and microdilution method in accordance with the guidelines of the European Committee on Antimicrobial Susceptibility Testing (EUCAST) [36] and the National Reference Centre for Susceptibility Testing in Poland (KORLD) [37]. The following reference strains were used to control the detection of drug resistance mechanisms: *Klebsiella pneumoniae* ATCC 700603 (ESBL+), *E. coli* ATCC 25922 (ESBL-), *Staphylococcus aureus* NCTC 12493 (MRSA+), *Enterococcus faecalis* ATCC 51299 (VAN B, HLAR), *Klebsiella pneumoniae* (KPC+), *Klebsiella pneumoniae* NCTC 13440 (MBL+), *E. coli* NCTC 13846 (colistin-resistant strain), *E. coli* ATCC 25922 (colistin-sensitive strain), and *Pseudomonas aeruginosa* ATCC 27853 (colistin-sensitive strain). 

ESBL was detected by double disc synergy test (DDST) with 30 µg ceftazidime and 30 µg cefotaxime at a distance of 2 cm (between centers) from the 20/10 µg amoxicillin/clavulanic acid disc. In cases of doubt, discs containing 10 µg of cefpodoxime or 30 µg of aztreonam were added. The positive test was evidenced by the enlargement of the growth inhibition zone around the ceftazidime or cefotaxime (cefpodoxime, aztreonam) disc on the side of the clavulanic acid disc. In order to detect ESBLs in AmpC producers, the test was performed simultaneously on Mueller Hinton Agar with the addition of cloxacillin 0.25 g/L. The cloxacillin test also served as the basis for the detection of the production of AmpC cephalosporinases. The study was performed for epidemiological purposes because according to the EUCAST guidelines, the cephalosporin susceptibility result was based on routine susceptibility testing by microdilution or disc diffusion. This method has been described and is recommended by experts of the National Reference Centre for Susceptibility Testing in Poland [38]. Susceptibility to colistin was tested by broth microdilution using the commercial MIC Colistin Mikrolatest (Erba Lacheme).

The analysis was focused on aetiological factors of bacterial infections, especially the incidence of MDR bacteria and mechanisms of antibiotic resistance. Microbiological monitoring was conducted in the accordance to regulations by the Polish Minister of Health. The source document presents the list of alarming microorganisms and guidelines on the registers for hospital infections and alarming microorganisms and reports on the current epidemiological situation of the hospital (last update on 1/22/21) [39]. Special attention was paid to the following mechanisms: bacteria producing AmpC-type beta-lactamases (AmpC), bacteria producing extended-spectrum beta-lactamases (ESBL), bacteria producing metallo-beta-lactamase (MBL), meticillin-resistant *Staphylococcus aureus* (MRSA), vancomycin-resistant *Enterococcus* (VRE), multidrug resistant non-fermenting gram-negative bacteria *Acinetobacter* spp., and *Pseudomonas aeruginosa* (resistant to carbapenems or other two classes of drugs or to polymyxins). Bacteria that did not meet the definition of multidrug resistant organisms (resistant to at least three groups of drugs), such as *Streptococcus pneumoniae* and *Clostridium perfringens*, were excluded from the analysis. Secondary analyses were aimed at identification of differences in the rate or aetiology of MDR bacterial infections between years and hospital departments of different medical specialty. Hospital-acquired infections were defined as diagnosed after 72 h of hospitalization according to the National Healthcare Safety Network (NHSN) microbiological monitoring definition (Multidrug-Resistant Organism & Clostridioides difficile Infection (MDRO/CDI) Module) [40].

Results are presented as absolute numbers and percentages. Differences between years and hospital departments were considered only if a minimum of 30 isolates was available for each MDR bacteria.

## 5. Conclusions

MDR bacteria are aetiological factors of a significant portion of infections in hospitalized patients. Production of ESBL is the most common mechanism of antibiotic resistance and should be regarded as one of the most urgent problems in clinical microbiology. The problem of drug resistance in hospitals requires constant monitoring and active measures to reduce the risk of MDR bacteria transmission.

## Figures and Tables

**Table 1:** Number of hospitalized patients and number of performed microbiological tests, including blood and urine tests in years 2017–2019 depending on department medical specialty.

Hospital Department	Number of Hospitalized Patients	Number of Microbiological Tests (Mean Number of Tests per Patient)	Number of Blood Cultures (Percent of All Microbiological Tests)	Number of Urine Cultures (Percent of All Microbiological Tests)
Urology	3805	4576 (1.20)	344 (7.5%)	3477 (76.0%)
Vascular Surgery	3283	559 (0.17)	60 (10.7%)	32 (5.7%)
General Surgery	5836	2293 (0.39)	234 (10.2%)	135 (5.9%)
Orthopaedic Surgery	5748	683 (0.12)	108 (11.9%)	77 (11.3%)
Laryngology	3742	362 (0.10)	44 9 (12.2%)	27 (7.5%)
Neurosurgery	2874	1014 (0.35)	391 (38.6%)	135 (13.3%)
Pediatric Surgery (2017 only)	815	43 (0.05)	3 (7.0%)	11 (25.6%)
Gynaecology	6150	1856 (0.30)	92 (5.0%)	174 (9.4%)
Obstetrics	9182	2345 (0.26)	115 (4.9%)	373 (15.9%)
Endocrinology	6006	634 (0.11)	92 (14.5%)	370 (58.4%)
Gastroenterology	7010	3073 (0.44)	486 (15.8%)	674 (21.9%)
Geriatrics (2018–2019 only)	320	17 (0.05)	0 (0%)	5 (29.4%)
Internal Diseases	9509	17,826 (1.87)	5851 (32.8%)	3799 (21.3%)
Cardiology	6166	1828 (0.30)	520 (28.4%)	282 (15.4%)
Neurology	4920	2300 (0.47)	733 (31.9%)	603 (26.2%)
Psychiatry	1661	510 (0.31)	160 (31.4%)	215 (42.2%)
Emergencies	56,513	998 (0.02)	141 (14.1%)	232 (23.2%)
Intensive Care—general	1054	3256 (3.09)	646 (19.8%)	501 (47.5%)
Intensive Care—cardiology	2292	625 (0.27)	305 (48.8%)	374 (59.8%)
Paediatrics	6048	10,704 (1.77)	3517 (32.9%)	2908 (27.2%)
Neonatology	6518	1124 (0.17)	371 (33.0%)	35 (3.1%)
Newborn Pathology	1077	2929 (2.72)	920 (31.4%)	50 (1.7%)
Total	150,529	58,789 (0.39)	15,082 (25.7%)	14,360 (24.4%)

**Table 2:** Etiological factors of infections diagnosed in different hospital departments.

Group of Hospital Departments	*S. aureus*	*E. faecium*	*E. faecalis*	*E. coli*	*Klebsiella* spp.	*Enterobacter* spp.	*P. aeruginosa*	*Acinetobacter* spp.
Intensive care	125 (25.4%)	23 (4.7%)	34 (6.9%)	68 (13.8%)	76 (15.4%)	26 (5.3%)	39 (7.9%)	86 (17.4%)
Emergencies	8 (9.5%)	1 (1.2%)	10 (11.9%)	23 (27.4%)	7 (8.3%)	2 (2.4%)	3 (3.6%)	0 (0%)
Urology	18 (2.5%)	13 (1.8%)	106 (15.0%)	351 (49.6%)	106 (15.0%)	44 (6.2%)	37 (5.2%)	19 (2.7%)
Other surgical departments *	303 (17.2%)	78 (4.4%)	245 (13.9%)	599 (34.0%)	174 (9.9%)	100 (5.7%)	113 (6.4%)	75 (4.3%)
Conservative treatment departments **	386 (9.8%)	204 (5.2%)	351 (8.9%)	1162 (29.6%)	582 (14.8%)	117 (3.0%)	218 (5.6%)	189 (4.8%)
Pediatric departments ***	66 (9.2%)	4 (0.6%)	40 (5.6%)	498 (69.6%)	65 (9.1%)	22 (3.1%)	15 (2.1%)	5 (0.7%)
Total	906 (11.8%)	323 (4.2%)	786 (10.2%)	2701 (35.1%)	1010 (13.1%)	311 (4.0%)	425 (5.5%)	374 (4.9%)

* Other surgical departments include Vascular Surgery, General Surgery, Orthopaedic Surgery, Laryngology, Neurosurgery, Pediatric Surgery, Gynaecology, Obstetrics. ** Conservative treatment departments include Endocrinology, Gastroenterology, Geriatrics, Internal Diseases, Cardiology, Neurology, Psychiatry. *** Pediatric departments include Pediatrics, Neonatology, Newborn Pathology.

**Table 3:** Multi-drug resistant bacteria as the etiological factor of infections.

	MRSA (among All *S. aureus* Cases)	VRE *E. faecium* (among All *E. faecium* Cases)	VRE *E. faecalis* (among All *E. faecalis* Cases)	ESBL *E. coli* (among All *E. coli* Cases)	AmpC *E. coli* (among All *E. coli* Cases)	ESBL *Klebsiella* (among All *Klebsiella* Cases)	MBL *Klebsiella* (among All *Klebsiella* Cases)	ESBL *Enterobacter* (among All *Enterobacter* Cases)	MDR *Acinetobacter* Spp.	MDR *P. aeruginosa* (among All *P. aeruginosa* Cases)
Total number of cases	262	184	69	359	14	418	100	67	250	60
The incidence of infections *	0.17%	0.12%	0.05%	0.24%	0.01%	0.28%	0.07%	0.04%	0.17%	0.04%
Percentage of hospital-acquired infections ** (n)	72.9% (191)	77.7% (143)	79.7% (55)	40.4% (145)	28.6% (4)	60.3% (252)	81.0% (81)	74.6% (50)	85.6% (214)	66.7% (40)
Rate of hospital-acquired to non-hospital-acquired infections	2.69	3.49	3.93	0.68	0.40	1.52	4.26	2.94	5.94	2.00

* Percentage of patients diagnosed with MDR bacterial infections among all hospitalized patients. ** Cases diagnosed after >72 h of hospitalization. AmpC—bacteria producing AmpC-type beta-lactamases; ESBL—bacteria producing extended-spectrum beta-lactamases; MBL—bacteria producing metallo-beta-lactamase; MDR—multidrug resistant bacteria; MRSA—methicyllin-resistant *Staphylococcus aureus*; VRE—vancomycin-resistant *Enterococcus*.

**Table 4:** The percentage of multidrug resistant bacteria diagnosed in different hospital departments. Minimal number of 30 isolates per each strain and each department, otherwise not reported.

Group of Hospital Departments	MRSA (among All *S. aureus* Cases)	ESBL *E. coli* (among All *E. coli* Cases)	ESBL Klebsiella (among All *Klebsiella* Cases)	MDR Acinetobacter (among All *Acinetobacter* Cases)
Intensive care	40.8%	n.a.	43.4%	80.2%
Urology	n.a.	13.7%	39.6%	n.a.
Other surgical departments *	33.7%	9.8%	35.1%	77.3%
Conservative treatment departments **	25.9%	18.9%	47.1%	58.7%

ESBL—bacteria producing extended-spectrum beta-lactamases; MDR—multidrug resistant bacteria; MRSA—methicyllin-resistant *Staphylococcus aureus*. * Other surgical departments include Vascular Surgery, General Surgery, Orthopaedic Surgery, Laryngology, Neurosurgery, Paediatric Surgery, Gynaecology, Obstetrics. ** Conservative treatment departments include Endocrinology, Gastroenterology, Geriatrics, Internal Diseases, Cardiology, Neurology, Psychiatry.

**Table 5:** The percentage (number of MDR infections to all infections) of infections caused by multidrug resistant bacteria depending on year of diagnosis.

Year	MRSA (among All *S. aureus* Cases)	VRE *E. faecium* (among All *E. faecium* Cases)	VRE *E. faecalis* (among All *E. faecalis* Cases)	ESBL *E. coli* (among All *E. coli* Cases)	AmpC *E. coli* (among All *E. coli* Cases)	ESBL *Klebsiella* (among All *Klebsiella* Cases)	MBL *Klebsiella* (among All *Klebsiella* Cases)	ESBL *Enterobacter* (among All *Enterobacter* Cases)	MDR *P. aeruginosa* (among All *P. aeruginosa* Cases)	MDR *Acinetobacter* (among All *Acinetobacter* Cases)
2017	25.1%	65.0%	10.5%	14.7%	0.8%	42.7%	7.9%	25.5%	12.7%	74.2%
2018	26.5%	58.3%	9.8%	14.8%	0.1%	33.7%	14.8%	20.0%	16.8%	61.7%
2019	34.5%	38.2%	5.9%	10.7%	0.6%	47.1%	7.8%	19.5%	13.5%	63.9%
Total	28.9%	56.7%	8.8%	13.3%	0.5%	41.4%	9.9%	21.5%	14.1%	66.8%

AmpC—bacteria producing AmpC-type beta-lactamases; ESBL—bacteria producing extended-spectrum beta-lactamases; MBL—bacteria producing metallo-beta-lactamase; MDR—multidrug resistant bacteria; MRSA—methicyllin-resistant *Staphylococcus aureus*; VRE—vancomycin-resistant *Enterococcus*.

## Data Availability

The data presented in this study are available upon request from the corresponding author. The data are not publicly available due to legal and privacy issues.
